# NPFFR2 Activates the HPA Axis and Induces Anxiogenic Effects in Rodents

**DOI:** 10.3390/ijms18081810

**Published:** 2017-08-21

**Authors:** Ya-Tin Lin, Yu-Lian Yu, Wei-Chen Hong, Ting-Shiuan Yeh, Ting-Chun Chen, Jin-Chung Chen

**Affiliations:** 1 Graduate Institute of Biomedical Sciences, Department of Physiology and Pharmacology, Chang Gung University, No. 259 Wenhwa 1st Road, Guishan, Taoyuan 333, Taiwan; yatin@mail.cgu.edu.tw; 2 Department of Biomedical Sciences, Chang Gung University, Taoyuan 333, Taiwan; lanszoie@hotmail.com.tw (Y.-L.Y.); weichen31137@hotmail.com (W.-C.H.); qszescasd@gmail.com (T.-C.C.); 3 Department of Medicine, Chang Gung University, Taoyuan 333, Taiwan; tinaabc2222@yahoo.com.tw; 4 Neuroscience Research Center, Chang Gung Memorial Hospital, No. 5, Fusing St., Guishan, Taoyuan 333, Taiwan; 5 Healthy Aging Research Center, Chang Gung University, Taoyuan 333, Taiwan

**Keywords:** neuropeptide FF (NPFF), NPFFR2, HPA axis, anxiety, PVN, CRF

## Abstract

Neuropeptide FF (NPFF) belongs to the RFamide family and is known as a morphine-modulating peptide. NPFF regulates various hypothalamic functions through two receptors, NPFFR1 and NPFFR2. The hypothalamic-pituitary-adrenal (HPA) axis participates in physiological stress response by increasing circulating glucocorticoid levels and modulating emotional responses. Other RFamide peptides, including neuropeptide AF, neuropeptide SF and RFamide related peptide also target NPFFR1 or NPFFR2, and have been reported to activate the HPA axis and induce anxiety- or depression-like behaviors. However, little is known about the action of NPFF on HPA axis activity and anxiety-like behaviors, and the role of the individual receptors remains unclear. In this study, NPFFR2 agonists were used to examine the role of NPFFR2 in activating the HPA axis in rodents. Administration of NPFFR2 agonists, dNPA (intracerebroventricular, ICV) and AC-263093 (intraperitoneal, IP), time-dependently (in rats) and dose-dependently (in mice) increased serum corticosteroid levels and the effects were counteracted by the NPFF receptor antagonist, RF9 (ICV), as well as corticotropin-releasing factor (CRF) antagonist, α-helical CRF(9-41) (intravenous, IV). Treatment with NPFFR2 agonist (AC-263093, IP) increased c-Fos protein expression in the hypothalamic paraventricular nucleus and induced an anxiogenic effect, which was evaluated in mice using an elevated plus maze. These findings reveal, for the first time, that the direct action of hypothalamic NPFFR2 stimulates the HPA axis and triggers anxiety-like behaviors.

## 1. Introduction

The founding member of the RFamide family, FMRF-amide, was originally isolated from the clam, *Macrocallista nimbosa*, and was reported to be a cardioexcitatory peptide [1]. Subsequently, two mammalian FMRF-amides were purified from bovine brain, and were named neuropeptide FF (NPFF, FLFQPQRF-NH_2_) and neuropeptide AF (NPAF, AGEGLSSPFWSLAAPQRF-NH_2_) [2]. NPFF is sometimes referred to as morphine modulating peptide, but it exhibits no significant binding affinity towards opiate receptors [3]. Two G protein-coupled receptors were identified as NPFF receptors, namely NPFFR1 and NPFFR2 (also called OT7TO22 and HLWAR77, respectively) [4,5,6]. Both of these receptors are coupled to G_i/o_ protein upon activation and are highly expressed in the central nervous system, predominantly in the posterior pituitary, thalamus and hypothalamus [3,7,8]. Both NPFFR1 and NPFFR2 are expressed in various subregions of the hypothalamus, including paraventricular nucleus (PVN), which is upstream of the hypothalamic-pituitary-adrenal (HPA) axis [7,8].

NPFF was then characterized in the context of the RFamide peptide family. There are five subtypes of mammalian RFamide peptides, including NPFF, RF-amide-related peptide (RFRP), pyroglutamylated RF-amide peptide (QRFP), prolactin releasing peptide (PrRP) and kisspeptin (KISS) [9]. Among these subtypes, NPFF-derived peptides include NPFF and NPAF, while Neuropeptide SF (NPSF, also known as RFRP-1) and Neuropeptide VF (NPVF, also known as RFRP-3) are NPVF-derived peptides [9,10,11]. Both NPFF- and NPVF-derived peptides show binding affinity with NPFF receptors. NPFF-derived peptides display higher affinity with NPFFR2 than NPFFR1, while NPVF-derived peptides display high affinity with NPFFR1 and poor affinity with NPFFR2 [12]. Initial studies of NPFF were mainly focused on its interaction with opioid signaling and the subsequent modulation of antinociceptive effects [3,13,14]. However, emerging evidence has emphasized the modulatory role of NPFF on hypothalamus-related functions, including cardiovascular and neuroendocrine regulation [11]. 

The HPA axis mediates physiological stress response. Activation of the HPA begins with stimulation of the PVN in the hypothalamus, which releases corticotropin-releasing factor (CRF) to activate pituitary corticotrophs. Upon CRF stimulation, adrenocorticotrophic hormone (ACTH) is released from the pituitary into circulation. ACTH stimulates the secretion of glucocorcorticoid from the adrenal cortex [15], which modulates a wide variety of physiological functions. The HPA axis is known to control metabolism, the immune system, the reproductive system, as well as growth and feeding [15,16]. Importantly, chronic stress and the hyperactivity of HPA axis are highly related to depressive and anxiety-like behaviors [17,18].

NPFF has been reported to regulate hypothalamic PVN neurons [11]. NPFF and NPVF disinhibit γ-aminobutyric acid-ergic (GABAergic) projections to the parvocellular PVN, thereby activating downstream neural circuits [19]. On the other hand, NPFF augments GABAergic neurotransmission to magnocellular PVN neurons, inhibiting the downstream release of vasopressin [20]. These opposing functions may be attributable to individual receptor activities. Other RF-amide peptides have also been reported to regulate the activity of HPA axis and increase the blood corticosteroid (CORT) levels, including NPAF, NPSF, kisspeptin-13 and RFRP-3 [21,22,23,24]. The stimulation of NPFFR1 was reported to enhance circulating CORT levels [24], while chronic activation of NPFFR2 leads to HPA axis dysfunction [25]. Continuous stimulation of NPFFR2 possibly disrupts the negative feedback pathway of the HPA axis since the expression of hippocampal glucocorticoid receptors was reduced while serum CORT level increased. Consequently, these mice displayed depressive and anxiety-like behaviors [25].

Nevertheless, direct observations that describe the function of NPFFR2 in regulating the HPA axis are lacking. In an attempt to pinpoint the role of NPFFR2 in HPA axis regulation, NPFFR2 was modulated using pharmacological approaches in rodents, and the effects on HPA axis activity were measured.

## 2. Results

### 2.1. Activation of NPFFR2 Stimulates the HPA Axis in Mice and Rats

**Figure 1:**
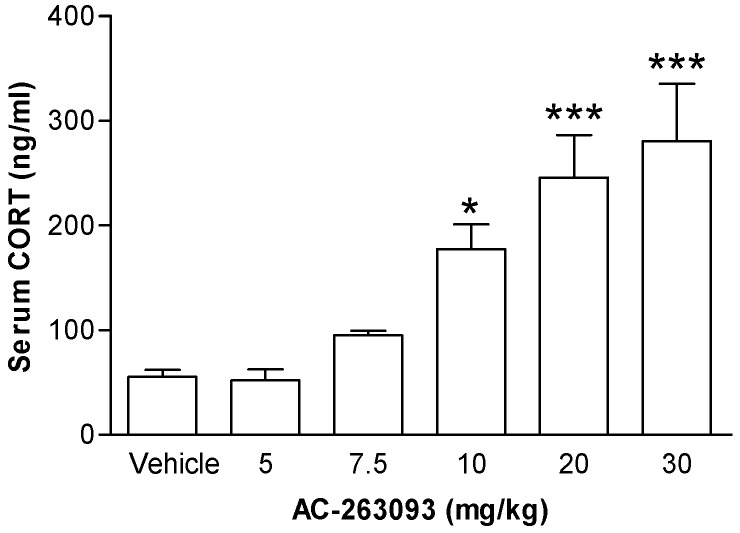
Effect of AC-263093 on the hypothalamic-pituitary-adrenal (HPA) axis in mice. Mice were intraperitoneal (IP) injected with vehicle or AC-263093 (5, 7.5, 10, 20, 30 mg/kg) and sacrificed after 1 h. Levels of serum corticosteroid (CORT) were measured by ELISA. Data are expressed as mean ± standard error of the mean (SEM) and were analyzed using a one-way ANOVA followed by Newman-Keuls post hoc tests. *, *p* < 0.05; ***, *p* < 0.001, compared to vehicle controls (*n* = 5 per group).

A NPFFR2 non-peptide agonist AC-263093 was used to stimulate the HPA axis. Mice were injected with AC-263093 (5, 7.5, 10, 20 or 30 mg/kg; intraperitoneal, IP) or vehicle and then sacrificed after 1 h by decapitation to collect trunk blood. Serum CORT was measured by a commercially available ELISA kit. The results showed that AC-263093 dose-dependently increased the serum CORT level, an effect that reached statistical significance at 10, 20 and 30 mg/kg compared to vehicle control (Figure 1). The data were analyzed by one-way ANOVA (F(5, 24) = 10.81, *p* < 0.0001) followed by Newman-Keuls post hoc tests. Another NPFFR2 agonist was used to confirm the observations made using AC-263093. Mice were injected with CFMHC (compound 3 in Ref. [26]) (30 mg/kg, IP) or vehicle and sacrificed after 1 h. The result showed that CFMHC increased serum CORT (Figure S3A).

**Figure 2:**
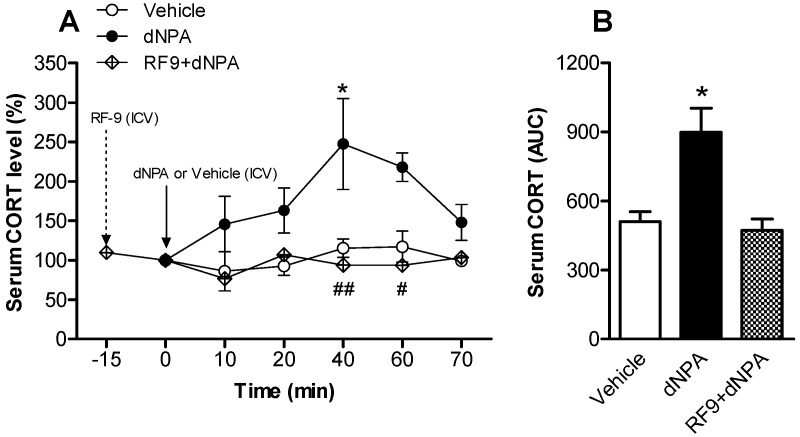
Time-dependent effects of treatment with dNPA on serum CORT in rats. Rats were injected with vehicle or dNPA (10 nmol, intracerebroventricular, ICV) and serum CORT was monitored up to 70 min following injection. (**A**) Levels of serum CORT were measured to indicate the activity of the HPA axis. Neuropeptide FF (NPFF) receptor antagonist RF9 (10 nmol, ICV) was administered 15 min prior to dNPA treatment. Data are expressed as mean ± SEM and were analyzed using a two-way ANOVA following by Bonferroni post hoc tests. * *p* < 0.05, compared dNPA to vehicle controls. # *p* < 0.05; ## *p* < 0.01, compared dNPA to RF9+dNPA group; (**B**) The results of area under curve (AUC) calculation. Data are expressed as mean ± SEM and were analyzed using a one-way ANOVA following by Bonferroni post hoc tests. * *p* < 0.05, compared dNPA to vehicle controls (*n* = 4–7 per group).

Further, conscious rats were utilized to monitor the changes of serum CORT at different time points following local administration of NPFFR2 agonist. The selective NPFFR2 agonist, dNPA, was administered to the lateral ventricle (10 nmol, intracerebroventricular, ICV) and blood samples were collected from the tail vein by intravenous (IV) catheter at 10 min intervals up to 70 min post-administration. The results showed that dNPA time-dependently elevated the serum CORT level, reaching a plateau 40 min after drug treatment (Figure 2A). Analysis with two-way ANOVA indicates that there were statistically significant effects of drug treatment (F(1, 5) = 17.28, *p* = 0.0001) and time (F(1, 5) = 2.442, *p* = 0.0443). There was no significant effect of the interaction between treatment and time. Bonferroni post hoc tests show the CORT level was significantly increased at 40 min post-drug administration when comparing dNPA-treated rat to vehicle controls (*p* < 0.05). The effect of dNPA was inhibited by the pre-treatment with NPFF receptor antagonist, RF9 (10 nmol, ICV), in the lateral ventricle 15 min prior to dNPA treatment (Figure 2A). Comparing the RF-9 pretreated and dNPA alone groups, a two-way ANOVA test revealed a statistically significant effect of drug treatment (F(1, 5) = 17.06, *p* = 0.0001). There was no significant effect from time, or the treatment and time interaction. Bonferroni post hoc tests indicate the CORT level was reduced and differences reached statistical significance at time points of 40 and 60 min. The area under curve (AUC) values further showed that the serum CORT was increased after treatment with dNPA and remained a vehicle-controlled level in rats pre-treated with RF9 (Figure 2B). A one-way ANOVA indicated that there was a statistically significant effect of drug treatment (F(2, 13) = 8.122, *p* = 0.0051). Rats treated with RF9 alone exhibited decreased serum CORT levels (Figure S2A), with a one-way ANOVA indicating a statistically significant effect of drug treatment (F(3, 20) = 5.697, *p* = 0.0055).

**Figure 3:**
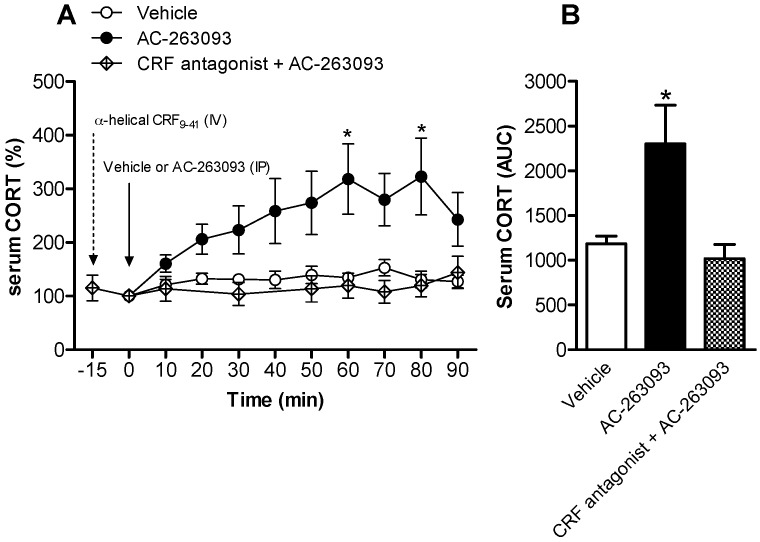
Time-dependent effects of treatment with AC-263093 on serum CORT in rats. Rats were injected with vehicle or AC-263093 (30 mg/kg, IP) and serum CORT was monitored for up to 90 min post-administration. (**A**) Levels of serum CORT were measured by ELISA. Corticotropin-releasing factor (CRF) antagonist α-helical CRF(9-41) (200 μg, intravenous, IV) was applied 15 min prior to AC-263093 treatment. Data are expressed as mean ± SEM and analyzed using a two-way ANOVA following by Bonferroni post hoc tests. * *p* < 0.05, compared AC-263093 to vehicle controls; (**B**) The results of AUC calculation. Data are expressed as mean ± SEM and were analyzed using a one-way ANOVA following by Bonferroni post hoc tests. * *p* < 0.05, compared dNPA to vehicle controls (*n* = 4–11 per group).

The activity of HPA axis was also evaluated after administration of the non-peptide NPFFR2 agonist, AC-263093. Rats were injected with AC-263093 (30 mg/kg, IP) and blood samples were collected from the tail vein of conscious animals, via IV catheter at 10 min intervals up to 90 min post-administration. The results showed that activation of NPFFR2 time-dependently enhanced serum CORT, with a peak level at 60 min after AC-263093 treatment (Figure 3A). A two-way ANOVA test indicates there was a statistically significant effect of AC-263093 treatment (F(1, 9) = 29.31, *p* < 0.0001), but there was no significant effect of time, or treatment and time interaction. Bonferroni post hoc tests show that the CORT level was increased at 60 and 80 min post-drug treatment when comparing the AC-263093-treated group to vehicle controls. The effect was inhibited by the pre-treatment of CRF antagonist α-helical CRF (9-41) (200 μg, IV) (Figure 3A). A two-way ANOVA test indicates there was a statistically significant effect of CRF antagonist treatment (F(1, 7) = 16.91, *p* < 0.0001), but no difference due to time or the interaction of treatment and time. The AUC calculations indicate that serum CORT was increased in the AC-263093-treated group, but was reduced to vehicle-controlled level in animals that were pre-treated with CRF antagonist (Figure 3B). The one-way ANOVA indicates that there was a statistically significant effect from drug treatment (F(2, 18) = 3.762, *p* = 0.0431). Rats treated with CRF antagonist alone showed decreased levels of serum CORT (Figure S2B). A one-way ANOVA indicated that there was a significant difference between drug treatments (F(3, 20) = 5.347, *p* = 0.0072).

### 2.2. NPFFR2 Agonist Induces Activity of Hypothalamic PVN Neurons in Mice

**Figure 4:**
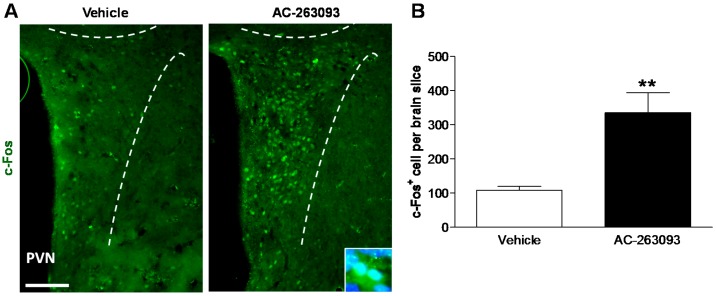
Effects of AC-263093 treatment on c-Fos expression in paraventricular nucleus (PVN) neurons. Mice were injected with vehicle or AC-263093 (30 mg/kg, IP) and sacrificed after 1 h. (**A**) Immunofluorescence staining was used to detect c-Fos expression in the PVN. Nuclei were counterstained with DAPI; (**B**) Quantification of c-Fos-positive cell numbers in the PVN. Histogram B shows the c-Fos immunoreactive neurons counts expressed as sum of bilateral frontal cross section surface areas of PVN. Data are expressed as mean ± SEM and were analyzed using an unpaired Student’s *t*-test. **, *p* < 0.01, compared to vehicle control (*n* = 4 per group). Scale bar = 100 μm.

Neuronal activity in the hypothalamic PVN was evaluated to verify the role of NPFFR2 on the HPA axis. The levels of c-Fos protein were measured 1 h after mice were treated with AC-263093 (30 mg/kg, IP) or vehicle. The results showed that c-Fos protein was upregulated in the hypothalamic PVN of NPFFR2 agonist-treated mice (Figure 4A). Quantifying the number of c-Fos-positive neurons within the PVN indicated that treatment with NPFFR2 agonist increased PVN activity (unpaired Student’s *t*-test, *t*(6) = 3.809, *p* = 0.0089) (Figure 4B). This finding demonstrates that stimulation of NPFFR2 enhances the activity of hypothalamic PVN neurons.

### 2.3. NPFFR2 Stimulation Induces Anxiety-Like Behavior

**Figure 5:**
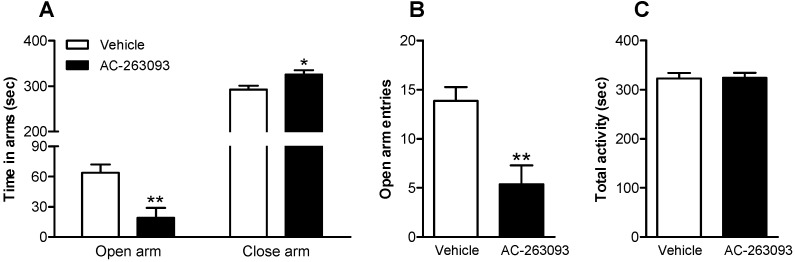
Effects of AC-263093 treatment on anxiety-like behavior. Mice were injected with AC-263093 (30 mg/kg, IP) 1 h prior to behavioral testing. Anxiety-like behavior was evaluated by the elevated plus maze (EPM). (**A**) Time that mice stayed in the open arms or closed arms; (**B**) Number of entries into open arms; (**C**) Total time spent with movement. Data are expressed as mean ± SEM and were analyzed using an unpaired Student’s *t*-test. *, *p* < 0.05; **, *p* < 0.01, compared to vehicle controls (*n* = 7–8 per group).

Anxiety-like behavior was monitored after 1 h, in mice treated with AC-263093 (30 mg/kg, IP). The elevated plus maze (EPM) was used to evaluate anxiety-like behavior. The duration in the open arm was reduced (unpaired Student’s *t*-test, *t*(13) = 3.406, *p* = 0.0047), while the duration in the closed arm was increased in AC-263093-treated mice, compared to vehicle controls (unpaired Student’s *t*-test, *t*(13) = 2.532, *p* = 0.0251) (Figure 5A). In addition, the number of open arm entries was decreased in mice treated with AC-263093 (unpaired Student’s *t*-test, *t*(13) = 3.489, *p* = 0.0040) (Figure 5B), but the administration of AC-263093 had no influence on the locomotor activity, as evaluated by total time spent with movement in the EPM (Figure 5C) as well as open field (Figure S1). These results were then confirmed using another NPFFR2 agonist, CFMHC (Figure S3). The findings indicate that activation of NPFFR2 induces anxiety-like behavior.

## 3. Discussion

In this study, we utilized a pharmacological approach to uncover the direct involvement of NPFFR2 in determining stress-dependent biochemical and behavioral changes in rodents. NPFFR2 agonists, dNPA or AC-263093, administered to mice and rats, evoked increased serum CORT levels in a dose- and time-dependent manner. The phenomenon was validated by reversal using the NPFF receptor antagonist, RF9. The NPFFR2 agonist-induced elevation of serum CORT was also inhibited by a CRF antagonist. Mice treated with NPFFR2 agonist showed anxiety-like behavior (assessed by EPM) and increased c-Fos expression in hypothalamic PVN neurons. These findings suggest that the stimulation of NPFFR2 within the PVN activates the HPA axis. This might act concomitantly with other limbic structures to trigger the anxiogenic behavior.

In this study, mice were used to evaluate the dose-response of NPFFR2 agonist-induced changes in serum CORT, c-Fos expression in PVN and anxiety-like behaviors. Blood samples were collected upon decapitation, precluding time course measurements on these animals. Because it was technically not feasible to insert tail vein catheters into mice, time-course studies on NPFFR2 agonist-induced changes of serum CORT were performed in rats. Interestingly, our results show that the responses of serum CORT to NPFFR2 agonist were comparable between both rodent species. Furthermore, dNPA (a peptide with poor blood-brain barrier penetration that was administered ICV) and AC-263093 (a small molecule, administered by IP injection) were similarly effective at enhancing the levels of circulating CORT. Together, these findings confirmed that NPFFR2 acts in the central nervous system of both rats and mice to regulate the HPA axis.

There is a growing body of evidence describing the involvement of RFamide peptides in controlling anxiety-related behaviors and HPA axis activity, but the roles for NPFFR1 and NPFFR2 remain unclear. Rats treated with NPAF, NPSF, kisspeptin-13 and RFRP-3 all show enhanced levels of circulating CORT [21,22,23,24]. With the exception of NPSF, these neuropeptides also induce anxiety-like behaviors. It was speculated that NPSF may exhibit relatively weak binding affinity to both NPFF receptors. Thus, the physiological effects of NPSF may be mediated through acid sensing ion channels rather than NPFF receptors [23,27]. Because NPAF and RFRP-3 exert different and complementary binding affinities toward NPFFR2 and NPFFR1 [10,28], their similar effects suggest that both receptors may be involved in the regulation of the HPA axis and relevant behavioral changes. The effects of RFRP-3 on the HPA axis were inhibited by a newly developed NPFF receptor antagonist GJ14 [24]. This antagonist displays higher affinity toward NPFFR1 (IC_50_, 21.08 nM) than NPFFR2 (IC_50_, 387.34 nM), suggesting that activation of brain NPFFR1 stimulates the HPA axis [24]. To address the role of NPFFR2 in HPA axis activation, we treated rats with the highly selective NPFFR2 agonist dNPA, administered directly into the lateral ventricle of conscious animals. dNPA time-dependently increased the level of serum CORT and this phenomenon was inhibited by intra-ventricular pre-treatment of the NPFFR antagonist, RF9. The effect of NPFFR2 activation was confirmed using another NPFFR2 agonist, AC-263093, which dose- and time-dependently enhanced serum CORT. These data support a positive regulatory role for NPFFR2 on the activity of the HPA axis. Another reported NPFFR2 agonist, CFMHC, was also used to confirm the effect of AC-263093. The results indicate the administration of CFMHC not only increases the level of serum CORT but also induces anxiety-like behavior. However, it should be noted that the selectivity of CFMHC between NPFFR1 and NPFFR2 is not high [26].

When we measured NPFFR2 agonist-induced changes in serum CORT, the rats were conscious and gently restrained by hand with cotton gloves. Prior to the day of the experiment, the experimental operator handled the rats daily, lightly restraining the animals by hand and gently touching their faces to reduce anxious behaviors. On the day of the experiment, rats were accustomed to the touch of the operator, and exhibited greatly reduced physical struggles and flinching behavior, compared to the initial acclimation sessions. With this experimental protocol, we were able to continuously monitor the drug effects without the influence of anesthesia. Despite our efforts to acclimate the animals, the potential for restraint-induced stress response was still of concern. According to our CORT ELISA measurements, the concentration of serum CORT was approximately 200 ng/mL at time zero. This relatively elevated level of serum CORT at the initiation of the experiment suggests that the effects of dNPA and AC-263093 were in addition to an underlying elevation of serum CORT from handling stress. However, in our experiments with mice, AC-263093 was clearly demonstrated to activate the HPA axis in the absence of additional stressors (Figure 1). On the other hand, the injection of CRF antagonist and RF9 reduced the levels of serum CORT. This result implies that experimental rats were under handling stress, since the inhibition of NPFFR2 could reduce the activity of HPA axis and stress responses.

RF9 was first synthesized in 2006 and was recognized as an NPFF receptor antagonist [29]. It had been reported to inhibit opioid-induced hyperalgesia, development of tolerance toward opioids, NPFF-induced hypothermia and lipopolysaccharide (LPS)-triggered fever in mice [29,30,31]. However, RF9 was recently been reported to act as an agonist of kisspeptin [32,33,34]. In this study, RF9 was used to inhibit HPA axis activity that was stimulated by dNPA. Although we observed a significant inhibitory effect on the action of NPFFR2 agonist dNPA, the possibility that RF9 produces this effect despite acting on other receptors cannot be excluded. 

NPFF and other RFamide neuropeptides were reported to modulate various functions of the hypothalamus, including cardiovascular and neuroendocrine regulation, energy consumption, food intake and reproduction [10,11]. Both NPFFR1 and NPFFR2 are highly expressed in hypothalamic nuclei [11] and may modulate these effects. Furthermore, NPFF was reported to regulate GABA neurotransmission within parvocellular and magnocellular PVN neurons, and in turn, to modulate downstream neural pathways [19,20]. The regulation of parvocellular PVN neurons was further shown to be due to a reduction of inhibitory postsynaptic currents, causing neuronal depolarization [19]. NPFF was also shown to activate the oxytocin-containing hypothalamic PVN neurons that project to the brainstem and increase arterial blood pressure [35]. Besides modulating GABA, NPFF may affect CRF in the PVN. CRF is synthesized and expressed in the parvocellular PVN neurons [36,37], where it acts as an upstream regulator of the HPA axis [15]. It was previously reported that NPFF-induced c-Fos expression is not predominantly colocalized with CRF containing neurons in the PVN [35]. However, comparing a recent published article with our IHC figures, we notice that AC compound-induced c-Fos proteins distribute similarly among the PVN subregions, including CRF, vasopressin and oxytocin-containing neurons [38]. Nevertheless, we observed that NPFFR2 agonist increased c-Fos expression in the PVN, and that a CRF inhibitor could attenuate NPFFR2 agonist-induced CORT release. The results suggest that NPFFR2 may inactivate PVN GABA neurons, and as such, it is possible that NPFFR2 evokes hypothalamic CRF release from parvocellular neurons. It was noted that PrRP also increases CRF neural activity in the PVN [39]. Interestingly, the effects of PrRP were suggested to be mediated through NPFFR2, since PrRP exhibits high binding affinity toward NPFFR2 [40,41]. Therefore, our study and others suggest that NPFFR2 plays a positive modulatory role in hypothalamic CRF neurons. It was noted that the c-Fos positive neurons were slightly more numerous in the vehicle control group (approximately 100 neurons per brain slice). This might be due to the 5% dimethyl sulfoxide (DMSO) that was contained in the vehicle, which had been reported to have predominated influence on neural activities [42,43,44,45]. The handling stress may also have an influence on the basal c-Fos cell counts; however, that phenomenon does not affect our main conclusion. 

NPFF receptor antagonists have been reported to confer anxiolytic effects. Treatment with RF9 reverses amphetamine withdrawal-induced anxiety-like behavior [46], and dansyl-PQRamide (a putative NPFF antagonist) reduces anxiety responses triggered by ethanol withdrawal [47]. We have recently reported that chronic stimulation of NPFFR2 triggers hyperactivity of the HPA axis and disrupts hippocampal feed-back regulation resulting in depression-like behaviors in mice [25]. These results suggest an anxiogenic effect from brain NPFFR2, as reported in the present study. However, another study showed that intra-ventral tegmental area (VTA) injection of NPFF prevents mild stress-induced locomotor activity [48]. The inconsistent regulation of NPFF in different aspects of stress response might be due to the involvement of different neural circuitries that impinge on the VTA and hypothalamus.

## 4. Materials and Methods

### 4.1. Animals

Sprague Dawley (SD) rats (250–300 g) and C57BL/6 (B6) mice (8–10 weeks old) were purchased from the National Laboratory Animal Center (Taipei, Taiwan) and acclimatized to the room with controlled temperature, air humidity and a 12 h day-night cycle (light on at 7:00 AM). Mice were housed 5–6 per cage and rats were housed 2–3 per cage, with food (Western Lab 7001, Orange, CA, USA) and water ad libitum. All the animals used in this study were male. Animal handling and drug treatments were performed in strict accordance with the NIH Guide for the Care and Use of Laboratory Animals. All procedures were approved by the Animal Care Committee of Chang-Gung University (CGU 13-014, approved on 1 April 2013).

### 4.2. NPFFR2 Agonists

Non-peptide NPFFR2 agonists, AC-263093 (2-[(3,4-dibromophenyl) methylene]-hydrazine-carboximidamide hydrochloride) and CFMHC (2-[[4-chloro-3-(trifluoromethyl)phenyl]methylene]-Hydrazinecarboximidamidehydrochloride) were used in current study [26]. The preparation of AC-263093 and CFMHC was described previously [25,49]. The drugs were dissolved in a vehicle solution consisting of DMSO: Tween 20: saline at a 1:2:17 ratio. Selective peptide NPFFR2 agonist dNPA (D.Asn-Pro-(N-Me)Ala-Phe-Leu-Phe-Gln-Pro-Gln-Arg-Phe-NH2) was commercially synthesized by Genemed Synthesis (San Antonio, TX, USA) and dissolved in 20% methanol with PBS, then diluted to 10% methanol before use.

### 4.3. Implantation and Cannulation in the Lateral Ventricle

Rats were anesthetized with pentobarbital (45 mg/kg, IP) and atropine (0.1 mg/kg, IP), and then placed into a stereotaxic instrument (David Kopf Instruments, Tujunga, CA, USA). The animals were implanted with a 23 G cannula positioned in the lateral ventricle at the coordinates: 0.8 mm posterior to the bregma, 1.4 mm lateral from the midline, and 3.7 mm ventral from the dura [50]. A stainless tube was left inside of cannula (0.5 mm protrusion) to prevent the cannula blocked by blood clog. After experiments, the brain was collected and sectioned by the rat brain matrices to validate the position of cannula using the anatomic microscope. Ampicillin (1 mg/kg, IP) was daily applied to prevent infection after the surgery. Rats were allowed to recover for 5 d before receiving ICV injections of either vehicle or dNPA.

### 4.4. Pharmacological Intervention Studies

Rats were anesthetized with 2% isoflurane and an IV catheter (24 G, Terumo, Laguna, Philippines) was inserted into the tail vein. The catheter was flushed with 150 μL Heparin solution (50 U/mL, China Chemical & Pharmaceutical Co., Taipei City, Taiwan) to prevent blood clotting. After removal from the isoflurane anesthesia, rats were gently restrained by hand with cotton gloves to reduce anxious behavior (gentle body movement was allowed). A rest time of 30 min elapsed prior to drug treatment. To reduce the stress responses, the experimental operator needs to remain in daily close contact with rats by holding, slightly restraining by hand and gently touching the faces of rats. The rats became much less anxious during experimental days. The cotton gloves were put into the home cages of experimental rats daily for 20 min to get used by rats and acquired the smell of rat bedding on the same time. A 30 min intervals after rats recovered from isoflurane anesthesia but prior to drug treatment was also important to reduce the stress-induced responses.

Two tubes of unused blood (50 μL) were collected within this rest period to ensure the smooth functioning of the IV catheter. After treatment with vehicle or AC-263093 (30 mg/kg, IP), approximately 80 μL blood was collected at 10 min intervals until 90 min had elapsed. In the CRF antagonist-pretreated group, α-helical CRF(9-41) (200 μg in 300 μL saline, Phoenix Pharmaceuticals, Burlingame, CA, USA) was injected into the tail vein 15 min prior to AC-263093 treatment (blood samples at 20 and 40 min post-injection of AC-263093 were not collected). In addition, NPFFR2 selective agonist dNPA was applied to stimulate the HPA axis. After ICV vehicle or dNPA (10 nmol) injection, approximately 80 μL blood was collected at 10 min intervals until 70 min had elapsed. In the NPFFR antagonist-pretreated group, RF9 (10 nmol, Tocris Bristol, UK) was injected (ICV) 15 min prior to dNPA treatment. Time zero indicates the time point immediately before the treatment of NPFFR2 agonists (AC-263093 or dNPA). Due to the individual difference between rats, the levels of serum CORT were normalized to percentage and time zero served as 100% in each rat.

### 4.5. Immunohistochemistry (IHC)

Mice were anesthetized with 45 mg/kg pentobarbital and then perfused with 4% paraformaldehyde after treatment with AC-263093 (30 mg/kg, IP) or vehicle for 1 h. The brain was then removed and cryoprotected with 20% sucrose in potassium phosphate buffer saline (KPBS) overnight at 4 °C. Brain tissues were sliced into 30 μm sections using a freezing microtome (LEICA CM3050S, Bannockburn, IL, USA). After rinsing with KPBS, sections were incubated in primary antibody (anti-c-Fos, 1:1000; Santa Cruz, CA, USA) in 2% normal serum/0.3% Triton X-100/KPBS at 4 °C for 24 h. Afterward, sections were incubated with 1:200 FITC-conjugated secondary antibody (FITC anti-rabbit antibody; Jackson ImmunoResearch, West Grove, PA, USA) for 1 h at room temperature. Sections were then incubated with 5 μg/mL 4′,6-diamidino-2-phenylindole (DAPI, Roche, Switzerland) for 5 min before mounting with glycerol. Immunofluorescent staining were observed by fluorescence microscopy (Olympus, Tokyo, Japan). The immunofluorescent signals were analyzed by ImageJ software (NIH, Bethesda, MD, USA). The result was presented as c-Fos^+^ cell number in one brain slice of bilateral PVN. The specificity of c-Fos antibody (sc-52, Santa Cruz, CA, USA) in mouse brain had been tested in a previous report using synthetic c-Fos control peptide [51].

### 4.6. CORT ELISA Assay

To obtain serum samples, blood was clotted at room temperature for 30 min and centrifuged at 15,000 rcf for 15 min. Serum CORT were analyzed by ELISA kits (Enzo Life Sciences, Farmingdale, NY, USA) according to the manufacturer’s protocols.

### 4.7. Elevated Plus Maze (EPM)

EPM was used to test anxiety-like behavior. The EPM chamber contains two open arms and two closed arms, and is raised 100 cm off the ground. Light intensities in open arms and close arms are between 80–85 Lux. A test mouse was placed on the middle of the elevated maze (face to the open arms) and movement was tracked by video recording for a session of 6 min. The time spent in open and closed arms, and the numbers of entries into open arms were calculated from the video recording replay. The total activity was also recorded as the total time spent in movement within 6 min. Mice were considered to have entered the arms once the middle part of their body passed the border of the arms.

### 4.8. Open Field Activity

Mice were transferred to an open field chamber (40 cm × 40 cm) 60 min after injection with AC-263093 (30 mg/kg, IP). The activity was then evaluated by tracking the body movement within 30 min (EthoVision, Noldus, Wageningen, The Netherlands). The results are presented as the distance that mice moved in every 5 min for a total session of 30 min.

### 4.9. Statistical Analysis

The effect of NPFFR2 agonist-induced CORT elevation in mice was analyzed by one-way ANOVA followed by Newman-Keuls post hoc tests (independent factors). The effect of NPFFR2 agonist-induced CORT elevation in rats was analyzed by the repeated measures two-way ANOVA followed by Bonferroni post hoc tests (dependent factor was level of serum CORT, independent factor was drug treatment). The following AUC was analyzed by one-way ANOVA with Bonferroni post hoc tests (independent factors). The Immunostaining assay and behavioral test were analyzed by the unpaired Student’s *t*-test (independent factors). All the statistical analyses were performed using GraphPad Prism 5 software with statistical significance set as *p* < 0.05.

### 4.10. Experimental Summary

The experimental details are summarized below (Table 1).

## 5. Conclusions

In summary, we demonstrate, for the first time, that NPFFR2 positively modulates the HPA axis via hypothalamic CRF. Our finding that NPFFR2 regulates stress-dependent neural pathways advances our understanding of neuroendocrine regulation and opens new avenues for probing the functions of NPFF.

## Figures and Tables

**Table 1:** The experimental summary.

Experiment in Figure	Subject	Experimental Groups	*n* Numbers
Figure 1	Mouse	Vehicle	5
		AC-263093	5
Figure 2	Rat	Vehicle	5
		dNPA	7
		RF9 + dNPA	4
		RF9 (Figure S2)	6
Figure 3	Rat	Vehicle	7
		AC-263093	11
		α-helical CRF(9-41) + AC-263093	4
		α-helical CRF(9-41) (Figure S2)	6
Figure 4	Mouse	Vehicle	4
		AC-263093	4
Figure 5	Mouse	Vehicle	7
		AC-263093	8

## Supplementary Materials

Supplementary materials can be found at www.mdpi.com/1422-0067/18/8/1810/s1.

## References

[1] Price D.A., Greenberg M.J. (1977). Structure of a molluscan cardioexcitatory neuropeptide. Science.

[2] Mulderry P.K., Ghatei M.A., Bishop A.E., Allen Y.S., Polak J.M., Bloom S.R. (1985). Distribution and chromatographic characterisation of CGRP-like immunoreactivity in the brain and gut of the rat. Regul. Pept..

[3] Yang H.Y., Tao T., Iadarola M.J. (2008). Modulatory role of neuropeptide FF system in nociception and opiate analgesia. Neuropeptides.

[4] Bonini J.A., Jones K.A., Adham N., Forray C., Artymyshyn R., Durkin M.M., Smith K.E., Tamm J.A., Boteju L.W., Lakhlani P.P. (2000). Identification and characterization of two G protein-coupled receptors for neuropeptide FF. J. Biol. Chem..

[5] Elshourbagy N.A., Ames R.S., Fitzgerald L.R., Foley J.J., Chambers J.K., Szekeres P.G., Evans N.A., Schmidt D.B., Buckley P.T., Dytko G.M. (2000). Receptor for the pain modulatory neuropeptides FF and AF is an orphan G protein-coupled receptor. J. Biol. Chem..

[6] Hinuma S., Shintani Y., Fukusumi S., Iijima N., Matsumoto Y., Hosoya M., Fujii R., Watanabe T., Kikuchi K., Terao Y. (2000). New neuropeptides containing carboxy-terminal RFamide and their receptor in mammals. Nat. Cell Biol..

[7] Gouarderes C., Quelven I., Mollereau C., Mazarguil H., Rice S.Q., Zajac J.M. (2002). Quantitative autoradiographic distribution of NPFF1 neuropeptide FF receptor in the rat brain and comparison with NPFF2 receptor by using [125I]YVP and [125I]EYF as selective radioligands. Neuroscience.

[8] Gouarderes C., Puget A., Zajac J.M. (2004). Detailed distribution of neuropeptide FF receptors (NPFF1 and NPFF2) in the rat, mouse, octodon, rabbit, guinea pig, and marmoset monkey brains: A comparative autoradiographic study. Synapse.

[9] Ayachi S., Simonin F. (2014). Involvement of mammalian RF-amide peptides and their receptors in the modulation of nociception in rodents. Front. Endocrinol..

[10] Findeisen M., Rathmann D., Beck-Sickinger A.G. (2011). RFamide peptides: Structure, function, mechanisms and pharmaceutical potential. Pharmaceuticals.

[11] Jhamandas J.H., Goncharuk V. (2013). Role of neuropeptide FF in central cardiovascular and neuroendocrine regulation. Front. Endocrinol..

[12] Liu Q., Guan X.M., Martin W.J., McDonald T.P., Clements M.K., Jiang Q., Zeng Z., Jacobson M., Williams D.L., Yu H. (2001). Identification and characterization of novel mammalian neuropeptide FF-like peptides that attenuate morphine-induced antinociception. J. Biol. Chem..

[13] Panula P., Kalso E., Nieminen M., Kontinen V.K., Brandt A., Pertovaara A. (1999). Neuropeptide FF and modulation of pain. Brain Res..

[14] Mouledous L., Mollereau C., Zajac J.M. (2010). Opioid-modulating properties of the neuropeptide FF system. Biofactors.

[15] Ulrich-Lai Y.M., Herman J.P. (2009). Neural regulation of endocrine and autonomic stress responses. Nat. Rev. Neurosci..

[16] Carrasco G.A., van de Kar L.D. (2003). Neuroendocrine pharmacology of stress. Eur. J. Pharmacol..

[17] Herman J.P., Tasker J.G. (2016). Paraventricular hypothalamic mechanisms of chronic stress adaptation. Front. Endocrinol..

[18] Dedic N., Chen A., Deussing J.M. (2017). The CRF family of neuropeptides and their receptors-mediators of the central stress response. Curr. Mol. Pharmacol..

[19] Jhamandas J.H., Simonin F., Bourguignon J.J., Harris K.H. (2007). Neuropeptide FF and neuropeptide VF inhibit GABAergic neurotransmission in parvocellular neurons of the rat hypothalamic paraventricular nucleus. Am. J. Physiol. Regul. Integr. Comp. Physiol..

[20] Jhamandas J.H., MacTavish D., Harris K.H. (2006). Neuropeptide FF (NPFF) control of magnocellular neurosecretory cells of the rat hypothalamic paraventricular nucleus (PVN). Peptides.

[21] Jaszberenyi M., Bagosi Z., Thurzo B., Foldesi I., Szabo G., Telegdy G. (2009). Endocrine, behavioral and autonomic effects of neuropeptide AF. Horm. Behav..

[22] Csabafi K., Jaszberenyi M., Bagosi Z., Liptak N., Telegdy G. (2013). Effects of kisspeptin-13 on the hypothalamic-pituitary-adrenal axis, thermoregulation, anxiety and locomotor activity in rats. Behav. Brain Res..

[23] Jaszberenyi M., Bagosi Z., Csabafi K., Palotai M., Telegdy G. (2014). The actions of neuropeptide SF on the hypothalamic-pituitary-adrenal axis and behavior in rats. Regul. Pept..

[24] Kim J.S., Brownjohn P.W., Dyer B.S., Beltramo M., Walker C.S., Hay D.L., Painter G.F., Tyndall J.D., Anderson G.M. (2015). Anxiogenic and stressor effects of the hypothalamic neuropeptide RFRP-3 are overcome by the NPFFR antagonist GJ14. Endocrinology.

[25] Lin Y.T., Liu T.Y., Yang C.Y., Yu Y.L., Chen T.C., Day Y.J., Chang C.C., Huang G.J., Chen J.C. (2016). Chronic activation of NPFFR2 stimulates the stress-related depressive behaviors through HPA axis modulation. Psychoneuroendocrinology.

[26] Gaubert G., Bertozzi F., Kelly N.M., Pawlas J., Scully A.L., Nash N.R., Gardell L.R., Lameh J., Olsson R. (2009). Discovery of selective nonpeptidergic neuropeptide FF2 receptor agonists. J. Med. Chem..

[27] Fukusumi S., Fujii R., Hinuma S. (2006). Recent advances in mammalian RFamide peptides: The discovery and functional analyses of PrRP, RFRPs and QRFP. Peptides.

[28] Yang H.Y., Iadarola M.J. (2006). Modulatory roles of the NPFF system in pain mechanisms at the spinal level. Peptides.

[29] Simonin F., Schmitt M., Laulin J.P., Laboureyras E., Jhamandas J.H., MacTavish D., Matifas A., Mollereau C., Laurent P., Parmentier M. (2006). RF9, a potent and selective neuropeptide FF receptor antagonist, prevents opioid-induced tolerance associated with hyperalgesia. Proc. Natl. Acad. Sci. USA.

[30] Fang Q., Wang Y.Q., He F., Guo J., Guo J., Chen Q., Wang R. (2008). Inhibition of neuropeptide FF (NPFF)-induced hypothermia and anti-morphine analgesia by RF9, a new selective NPFF receptors antagonist. Regul. Pept..

[31] Wang Y.Q., Wang S.B., Ma J.L., Guo J., Fang Q., Sun T., Zhuang Y., Wang R. (2011). Neuropeptide FF receptor antagonist, RF9, attenuates the fever induced by central injection of LPS in mice. Peptides.

[32] Maletinska L., Ticha A., Nagelova V., Spolcova A., Blechova M., Elbert T., Zelezna B. (2013). Neuropeptide FF analog RF9 is not an antagonist of NPFF receptor and decreases food intake in mice after its central and peripheral administration. Brain Res..

[33] Min L., Leon S., Li H., Pinilla L., Carroll R.S., Tena-Sempere M., Kaiser U.B. (2015). RF9 Acts as a KISS1R Agonist In Vivo and In Vitro. Endocrinology.

[34] Sahin Z., Canpolat S., Ozcan M., Ozgocer T., Kelestimur H. (2015). Kisspeptin antagonist prevents RF9-induced reproductive changes in female rats. Reproduction.

[35] Jhamandas J.H., MacTavish D. (2003). Central administration of neuropeptide FF causes activation of oxytocin paraventricular hypothalamic neurones that project to the brainstem. J. Neuroendocrinol..

[36] Swanson L.W., Sawchenko P.E., Lind R.W. (1986). Regulation of multiple peptides in CRF parvocellular neurosecretory neurons: Implications for the stress response. Prog. Brain Res..

[37] Ludwig M., Leng G. (2006). Dendritic peptide release and peptide-dependent behaviours. Nat. Rev. Neurosci..

[38] Romanov R.A., Alpar A., Zhang M.D., Zeisel A., Calas A., Landry M., Fuszard M., Shirran S.L., Schnell R., Dobolyi A. (2015). A secretagogin locus of the mammalian hypothalamus controls stress hormone release. EMBO J..

[39] Yamada T., Mochiduki A., Sugimoto Y., Suzuki Y., Itoi K., Inoue K. (2009). Prolactin-releasing peptide regulates the cardiovascular system via corticotrophin-releasing hormone. J. Neuroendocrinol..

[40] Engstrom M., Brandt A., Wurster S., Savola J.M., Panula P. (2003). Prolactin releasing peptide has high affinity and efficacy at neuropeptide FF2 receptors. J. Pharmacol. Exp. Ther..

[41] Ma L., MacTavish D., Simonin F., Bourguignon J.J., Watanabe T., Jhamandas J.H. (2009). Prolactin-releasing peptide effects in the rat brain are mediated through the Neuropeptide FF receptor. Eur. J. Neurosci..

[42] Nakahiro M., Arakawa O., Narahashi T., Ukai S., Kato Y., Nishinuma K., Nishimura T. (1992). Dimethyl sulfoxide (DMSO) blocks GABA-induced current in rat dorsal root ganglion neurons. Neurosci. Lett..

[43] Nasrallah F.A., Garner B., Ball G.E., Rae C. (2008). Modulation of brain metabolism by very low concentrations of the commonly used drug delivery vehicle dimethyl sulfoxide (DMSO). J. Neurosci. Res..

[44] Soltani N., Mohammadi E., Allahtavakoli M., Shamsizadeh A., Roohbakhsh A., Haghparast A. (2016). Effects of dimethyl sulfoxide on neuronal response characteristics in deep layers of rat barrel cortex. Basic Clin. Neurosci..

[45] Sawada M., Sato M. (1975). The effect of dimethyl sulfoxide on the neuronal excitability and cholinergic transmission in Aplysia ganglion cells. Ann. N. Y. Acad. Sci..

[46] Kotlinska J.H., Gibula-Bruzda E., Koltunowska D., Raoof H., Suder P., Silberring J. (2012). Modulation of neuropeptide FF (NPFF) receptors influences the expression of amphetamine-induced conditioned place preference and amphetamine withdrawal anxiety-like behavior in rats. Peptides.

[47] Kotlinska J., Pachuta A., Bochenski M., Silberring J. (2009). Dansyl-PQRamide, a putative antagonist of NPFF receptors, reduces anxiety-like behavior of ethanol withdrawal in a plus-maze test in rats. Peptides.

[48] Cador M., Marco N., Stinus L., Simonnet G. (2002). Interaction between neuropeptide FF and opioids in the ventral tegmental area in the behavioral response to novelty. Neuroscience.

[49] Lin Y.T., Kao S.C., Day Y.J., Chang C.C., Chen J.C. (2016). Altered nociception and morphine tolerance in neuropeptide FF receptor type 2 over-expressing mice. Eur. J. Pain..

[50] Herman J.P., Watson S.J., Paxinos G., Watson C. (1986). The Rat Brain in Stereotaxic Coordinates.

[51] Gaszner B., Kormos V., Kozicz T., Hashimoto H., Reglodi D., Helyes Z. (2012). The behavioral phenotype of pituitary adenylate-cyclase activating polypeptide-deficient mice in anxiety and depression tests is accompanied by blunted c-Fos expression in the bed nucleus of the stria terminalis, central projecting Edinger-Westphal nucleus, ventral lateral septum, and dorsal raphe nucleus. Neuroscience.

