# Supplementary material for: NPFFR2 Activates the HPA Axis and Induces Anxiogenic Effects in Rodents
Source: Int J Mol Sci. 2017 Aug 21;18(8):1810. doi: 10.3390/ijms18081810 (PMC5578197; doi:10.3390/ijms18081810)
Supplement: Supplementary file 1 [file ijms-18-01810-s001.pdf]

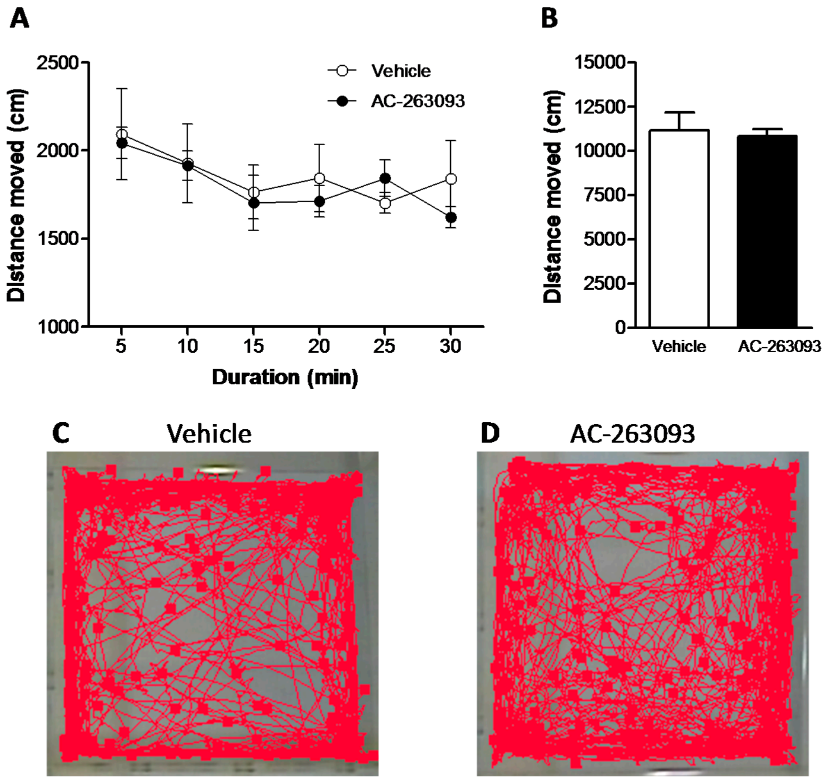

Figure S1. Effect of acute AC-263093 treatment on locomotor activity in mice. Mice were injected with AC-263093 (30 mg/kg, IP) 60 min prior to the behavioral test. The activity was evaluated by tracking the mice movement in 30 min (EthoVision, Noldus, The Netherlands). (**A**) Distance mice moved in every 5 min. (**B**) Total distance mice moved; (**C**,**D**) Representative mouse tracks after injection with vehicle or AC-263093. Data are expressed as mean ± S.E.M. and were analyzed using an unpaired Student's *t*-test (*N* = 4 per group).

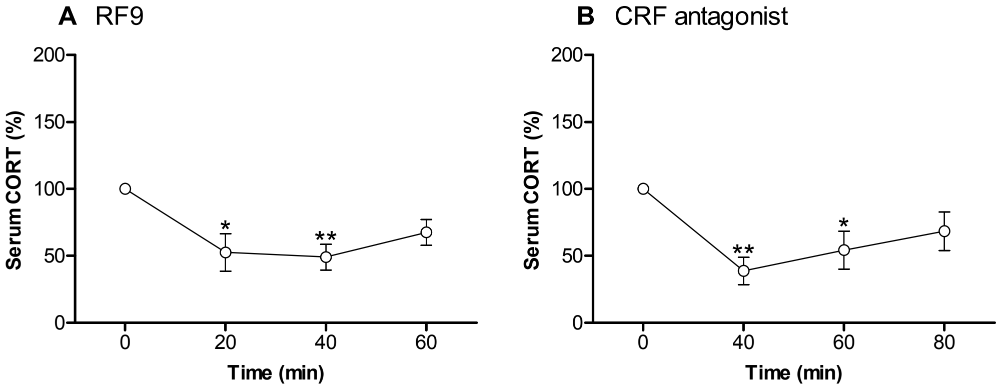

Figure S2. Time-dependent effect of RF9 or CRF antagonist on serum CORT levels in rats. Rats were injected with (**A**) RF9 (10 nmol, ICV) or (**B**) CRF antagonist α-helical CRF9-41 (200 μg, IV) and serum CORT was monitored for up to 80 min post-drug administration. Data are expressed as mean ± S.E.M and were analyzed using one-way ANOVA. ($N = 6$ per group). *, $p < 0.05$; **, $p < 0.01$, compared to time zero.

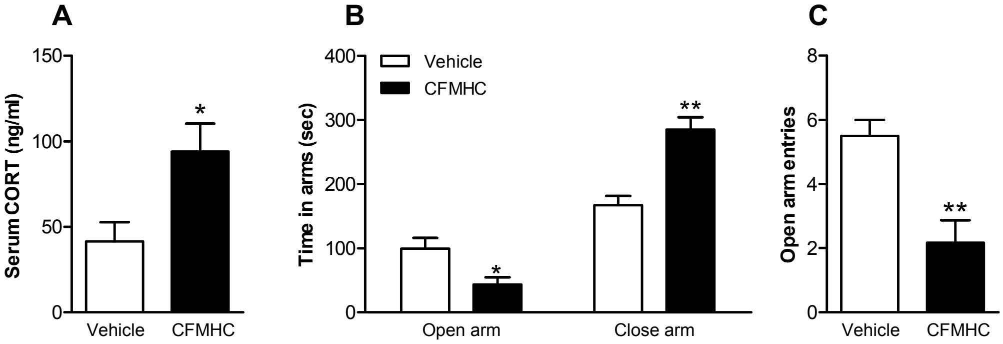

Figure S3. Effect of acute CFMHC treatment on serum CORT level and anxiety-like behaviors. Mice were injected with CFMHC (30 mg/kg, IP) 60 min prior to behavioral testing or serum collection. (**A**) Levels of serum CORT were measured to indicate the activity of the HPA axis. Anxiety-like behavior was evaluated by elevated plus maze; (**B**) Time that mice stayed in open versus closed arms; (**C**) Number of entries in open arms. Data are expressed as mean ± S.E.M. and were analyzed using an unpaired Student's *t*-test. *, $p < 0.05$; **, $p < 0.01$, compared to vehicle controls ($N = 4$-6 per group).
